# Comparative Biochemical, Structural, and Functional Analysis of Recombinant Phospholipases D from Three *Loxosceles* Spider Venoms

**DOI:** 10.3390/ijms241512006

**Published:** 2023-07-26

**Authors:** Hanna Câmara da Justa, Jorge Enrique Hernández González, Larissa Vuitika, Ricardo Barros Mariutti, Pedro Augusto Martinho Magnago, Fábio Rogério de Moraes, Andrea Senff-Ribeiro, Luiza Helena Gremski, Raghuvir Krishnaswamy Arni, Silvio Sanches Veiga

**Affiliations:** 1 Department of Cell Biology, Federal University of Paraná (UFPR), Curitiba 81531-980, Brazil; hannajusta@gmail.com (H.C.d.J.); vuitika@usp.br (L.V.); pedroaugusto.biotec@gmail.com (P.A.M.M.); senffribeiro@gmail.com (A.S.-R.); luizagremski@ufpr.br (L.H.G.); 2 Department of Physics, Multi-User Center for Biomolecular Innovation, State University of São Paulo (UNESP), São Paulo 05315-970, Brazil; jorge.hernandez@unesp.br (J.E.H.G.); ricardomariutti@yahoo.com.br (R.B.M.); fabio.moraes@unesp.br (F.R.d.M.); raghuvir.arni@unesp.br (R.K.A.); 3 Department of Immunology, Institute of Biomedical Sciences IV, University of São Paulo (ICB-IV/USP), São Paulo 05508-000, Brazil

**Keywords:** *Loxosceles intermedia*, *L. gaucho*, *L. laeta*, brown spider, venom, phospholipase D

## Abstract

Spiders of *Loxosceles* genus are widely distributed and their venoms contain phospholipases D (PLDs), which degrade phospholipids and trigger inflammatory responses, dermonecrosis, hematological changes, and renal injuries. Biochemical, functional, and structural properties of three recombinant PLDs from *L. intermedia*, *L. laeta,* and *L. gaucho*, the principal species clinically relevant in South America, were analyzed. Sera against *L. gaucho* and *L. laeta* PLDs strongly cross-reacted with other PLDs, but sera against *L. intermedia* PLD mostly reacted with homologous molecules, suggesting underlying structural and functional differences. PLDs presented a similar secondary structure profile but distinct melting temperatures. Different methods demonstrated that all PLDs cleave sphingomyelin and lysophosphatidylcholine, but *L. gaucho* and *L. laeta* PLDs excelled. *L. gaucho* PLD showed greater “in vitro” hemolytic activity. *L. gaucho* and *L. laeta* PLDs were more lethal in assays with mice and crickets. Molecular dynamics simulations correlated their biochemical activities with differences in sequences and conformations of specific surface loops, which play roles in protein stability and in modulating interactions with the membrane. Despite the high similarity, PLDs from *L. gaucho* and *L. laeta* venoms are more active than *L. intermedia* PLD, requiring special attention from physicians when these two species prevail in endemic regions.

## 1. Introduction

Spiders of the *Loxosceles* genus, popularly known as brown spiders, violin spiders, or recluse spiders [1,2,3,4], are cosmopolitan and represent a public health problem, especially in South American countries. In Brazil, for instance, 30,112 accidents were reported between 2018 and 2021 [5]. Currently, 14 *Loxosceles* species have been identified in Brazil [6], and among them, three stand out for causing the most accidents: *L. intermedia*, *L. laeta,* and *L. gaucho* [3,4,7]. The set of clinical manifestations that may develop after a *Loxosceles* bite, referred to as Loxoscelism [3,4,8], causes dermonecrosis, edema, and intense inflammatory response at the bite site and in the neighboring regions, which are the principal indications of cutaneous loxoscelism [2,3,4,8]. Severe cases may include hematological disturbances, which are mainly related to intravascular hemolysis and acute renal failure, which are the leading causes of death associated with Loxoscelism [2,3,4,8].

Although the venoms of these spiders consist of a range of proteins and peptides, the action of the whole venom can be better understood when the activity of phospholipases-D (PLDs) is examined as these toxins reproduce most of the effects observed in patients bitten by Brown spiders [9,10,11,12,13]. There are multiple isoforms of PLDs in the venoms of spiders from the Sicariidae family, which includes *Loxosceles* and *Sicarius* genera [14]. These toxins possess molecular masses of 32–35 kDa and are highly expressed in *Loxosceles* venom glands [15]. The amino acid sequence identities of Brown spider PLD isoforms range from 55 to 99% [16,17,18]. Crystallographic and systematic mutational/functional investigations have identified key amino acid residues involved in catalysis (H12 and H47), metal-ion binding (E32, D34, and D91), substrate recognition (K93, Y228, and W230), and stabilization of the flexible loop (C53 and C 201) [16,17,19,20,21].

The best-characterized substrates of these enzymes are sphingomyelin and lysophosphatidylcholine (1-lyso-2-acyl-phosphatidylcholine, 1-LysoPC) [22,23,24]. The hydrolysis of lysoglycerophospholipids by *Loxosceles* PLDs, such as lysophosphatidylcholine, lysophosphatidylinositol, lysophosphatidylethanolamine, lysophosphatidylserine, and lyso-platelet-activating factor (LPAF), results in the formation of choline and lysophosphospholipid substrates or the corresponding cyclic forms [22,23,24,25,26,27]. Ceramide 1-phosphate (C1P) and Lysophosphatidic acid (LPA) are bioactive lipid mediators implicated in the inflammatory response induced by *Loxosceles* PLDs.

Brown spider PLDs are classified into two structural groups based on the number of disulfide bonds and on the sphingomyelinase activity [19]. Class I PLDs are represented by enzymes that possess a single disulfide bridge formed between C51 and C57 and possess an extended flexible loop. Class II PLDs contain an additional disulfide bridge between C53 and C201, which links the flexible loop to the catalytic loop. Based on the ability to cleave sphingomyelin, these enzymes are further subdivided into two subclasses referred to as IIa (active PLDs) and IIb (less active PLDs) [19]. These differences may be associated with the distinct orientations of the substrate in the catalytic cleft that affects the affinity [18,19,20,21]. *Loxosceles* PLDs can be further classified in α- and β-clades, where α-clade members present high catalytic activity against sphingomyelin, whereas the activity of β-clade members is lower and is more variable [27].

We compare the functional and structural aspects of recombinant PLDs from the venoms of the three main species encountered in South America (*L. intermedia, L. laeta,* and *L. gaucho*) and correlate their biochemical and biological activities with their structural features. These analyses may help to understand the differences in the toxicity among these venoms and improve the alternatives to treat Loxoscelism.

## 2. Results

### 2.1. Amino Acid Sequence Comparison of PLDs

**Figure 1:**
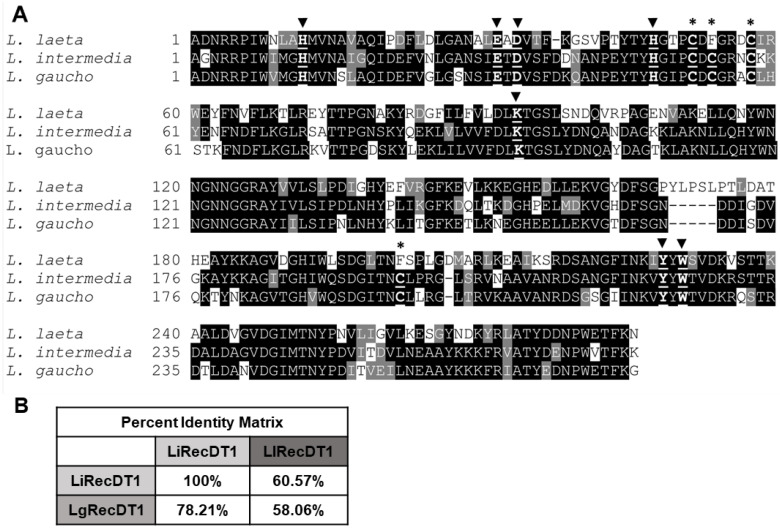
Comparison of the mature cDNA-deduced amino acid sequences of LiRecDT1 (GenBank: DQ218155.1), LgRecDT1 (GenBank: JX866729.1), and LlRecDT1 (GenBank: AY093599.1). (**A**) Multiple amino acid alignment of PLDs highlights the conserved regions in black, conservative substitutions in gray, and non-conservative substitutions in white. Arrowheads indicate conserved amino acid residues that form the catalytic pocket, that are also underlined. Asterisks indicate cysteine residues involved in forming disulfide bridges. Sequences were aligned using multiple sequence comparison by log-expectation (MUSCLE). (**B**) Percent identity matrix showing similarities among PLDs. Values were calculated by MUSCLE.

The mature cDNA sequences of LiRecDT1, LlRecDT1, and LgRecDT1 have 843, 858, and 843 base pairs, respectively, and their putative ORFs (open reading frames) code 281, 286, and 281 amino acid residues, respectively. The molecular masses and theoretical isoelectric points (pI) were calculated using the ProtParam tool and indicated molecular masses of 33.6 kDa, 34.4 kDa, and 33.8 kDa and isoelectric points of 7.7, 6.2, and 6.2 for LiRecDT1, LlRecDT1, and LgRecDT1, respectively. Multiple amino acid alignments indicated sequence identities ranging from 58.06% to 78.21% (Figure 1B) and confirmed that all the amino acid residues implicated in the enzymatic activity are conserved as highlighted in Figure 1A. As observed by the cysteine residues, LlRecDT1 belongs to Class I PLDs and LiRecDT1 and LgRecDT1 to Class II PLDs.

### 2.2. Immunological Relationship of Phospholipases D

**Figure 2:**
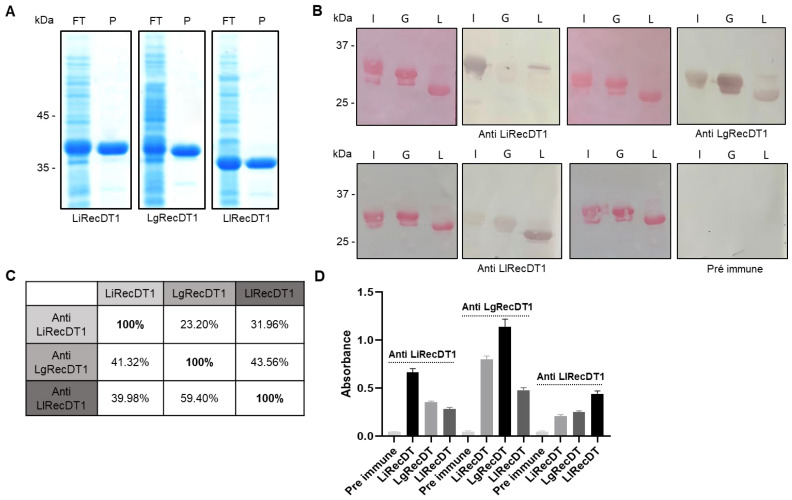
Results of the purification and antigenic characteristics of recombinant PLDs. (**A**) Expression and purification of recombinant toxins as analyzed by 12.5% Coomassie Blue stained reducing SDS-PAGE gels. Lanes FT (final time) depict *E. coli* BL21(DE3)pLysS cells after 3.5 h of induction with 0.05 mM IPTG. Lanes P present purified recombinant proteins from *L. intermedia* (LiRecDT1), *L. gaucho* (LgRecDT1), and *L. laeta* (LlRecDT1). Molecular protein mass standards are indicated on the left. (**B**) Purified recombinant toxins (5 µg) were submitted to SDS-PAGE transferred onto nitrocellulose membranes and stained with Ponceau S. Alternatively, membranes were incubated with polyclonal sera against LiRecDT1, LlRecDT1, or LgRecDT1. Pre-immune serum was used as a control for antibody specificity. I: LiRecDT1; L: LlRecDT1; G: LgRecDT1. (**C**) Densitometry of immune WB reactions showed in (**B**) analyzed by Image J software 1.54f. (**D**) ELISA was performed by coating with 20 μg/mL of purified recombinant toxins and incubating the plates with antibodies against LiRecDT, LgRecDT, and LlRecDT (1:4000).

Immunological studies of LiRecDT1, LlRecDT1, and LgRecDT1, expressed in *E. coli* using Western blotting (WB) (Figure 2B) and the enzyme-linked immunosorbent assay (ELISA) (Figure 2D), were performed to evaluate the antigenic cross-reactivity among the PLDs of three different venoms, normalized by protein concentration, using polyclonal antisera raised against LiRecDT1, LlRecDT1, and LgRecDT1 obtained under the same experimental conditions. The immune WB reactions were quantified by densitometric analysis of the positive bands (Figure 2C) and demonstrated that all antibodies reacted to the protein used in immunizations, but also reacted to the other toxins, demonstrating antigenic cross-reactivity and similar epitopes among recombinant PLDs of different species. The serum raised against *L. gaucho* PLD strongly cross-reacted with the other PLDs in both WB and ELISA assays, suggesting that LgRecDT1 has similar antigenic determinants compared to LiRecDT1 and LlRecDT1 (Figure 2B–D). Despite the significant amino acid identity among these PLDs, these results suggest structural and functional differences between these molecules and indicate that LgRecDT1 has a greater number of linear and conformational antigenic determinants (WB and ELISA).

### 2.3. Biophysical Properties of PLDs

**Figure 3:**
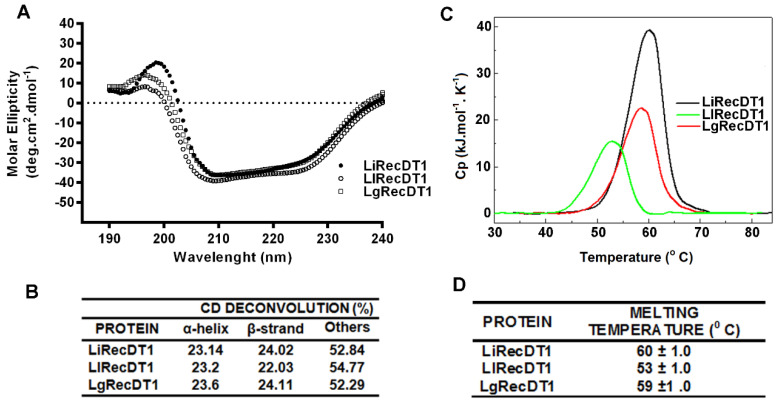
CD spectra and thermal stability of recombinant Brown spider phospholipases D. (**A**,**B**) Superposition of the CD spectra obtained by samples diluted in phosphate buffer at 20 °C and analyzed in a Jasco J-810 spectropolarimeter. Deconvolution was calculated using a K2D3 bioinformatic tool. (**C**,**D**) Thermal stability comparison of Brown spider phospholipases D (LiRecDT1, LlRecDT1, and LgRecDT1). DSC thermogram recorded for toxins at a scan rate of 1.0 °C/min in a temperature range of 20–90 °C using a phosphate buffer.

The secondary structures of recombinant PLDs were analyzed by circular dichroism spectroscopy (CD) and exhibited very similar spectra (Figure 3A). As expected, the percentages of secondary structures calculated by using the K2D3 bioinformatics software indicated that the three toxins possessed similar structures (23% α-helix and 22–24% β-sheet) (Figure 3B). 

The thermal stabilities of recombinant PLDs were investigated by differential scanning calorimetry. It was observed that the toxins are thermostable; however, they show minor differences in their melting temperatures (T_m_) during their irreversible unfolding process (Figure 3C,D). LiRecDT1 (T_m_ ~ 60 °C) was the most thermally stable, followed by LgRecDT1 (~58 °C) and LlRecDT1 (~53 °C). The initiation of the thermal denaturation processes for LiRecDT1 and LgRecDT1 occur at about 50 °C, whereas for LlRecDT1 it starts at a 7 °C lower temperature.

### 2.4. Enzymatic Activity of Recombinant PLDs

**Figure 4:**
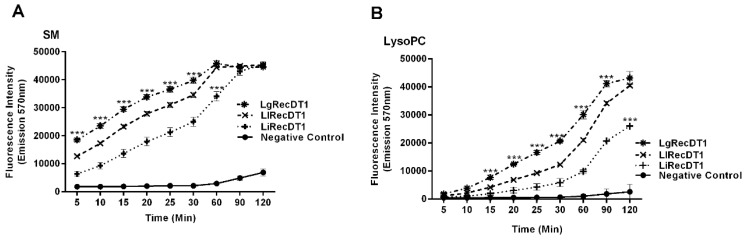
Enzymatic activity of recombinant PLDs upon SM and LysoPC measured by Amplex Red^TM^ assay. (**A**) Enzymatic activity of LiRecDT1, LlRecDT1, and LgRecDT1 upon SM depicted in a time-dependent analysis. (**B**) Enzymatic activity of LiRecDT1, LlRecDT1, and LgRecDT1 on LysoPC depicted in a time-dependent analysis. For both substrates, the product of reaction (choline) was fluorometrically measured by excitation at 540 nm and emission at 570 nm. Negative control was performed by incubation of Amplex Red reagent mixture without PLDs. The values represent average ± SEM of five independent experiments carried out in triplicate (*** *p <* 0.001). Statistical analyses compared the values corresponding to LgRecDT1 with those from LiRecDT1 and LlRecDT1.

The enzymatic activities for the recombinant PLDs were performed using two different lipid substrates, sphingomyelin (SM) and lysophosphatidylcholine (LysoPC), which are phospholipids that are present in cell membrane surfaces and are the targets for brown spider venom PLDs [4,15]. The cleavage products were measured by three different and complementary methods: (1) Amplex Red^TM^ assay, which evaluates the choline formation after enzyme degrading activity upon substrates, (2) HPTLC (high-performance thin-layer chromatography), and (3) NMR (nuclear magnetic resonance spectroscopy), which detect the cleaving activity of substrates and generated products following PLDs activity. The Amplex Red assay showed that all recombinant PLDs tested are able to cleave SM and LysoPC in a time-dependent manner (Figure 4A,B). From these results, it is possible to conclude that the action of the three toxins upon SM is faster than upon LysoPC. In addition, LgRecDT1 exhibits the highest catalytic activity among the three PLDs, on either SM or LysoPC, whereas LiRecDT1 from *L. intermedia* shows the lowest activity among the tested toxins.

**Figure 5:**
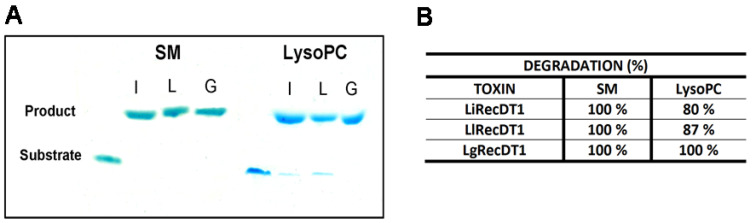
Enzymatic activity of recombinant PLDs upon SM and LysoPC analyzed by HPTLC. (**A**) The bands of substrates and products formed after incubation with LiRecDT1 (letter I), LlRecDT1 (letter L), and LgRecDT1 (letter G) and analyzed in silica gel plates are depicted after reaction with molybdenum blue. (**B**) Band intensities for substrates and generated products were estimated densitometrically and the percentages of degradation were normalized on the basis of the intensity of the bands from untreated substrates (considered 100%).

In addition, to confirm the distinct enzymatic activities of PLDs upon specific phospholipids, the cleavage of SM or LysoPC by toxins was also analyzed by HPTLC, which enables the detection of substrates and generated products during the experiment. The intensity of the substrate bands prior to PLDs treatments was reduced and the bands of generated products increased after 60 min of incubation with toxins. The results confirmed that all toxins display enzymatic activity upon both substrates, but prefer SM to LysoPC, as SM, but not LysoPC, was completely degraded at the end of the assay with the three PLDs (Figure 5A). In addition, the more intense activity of LgRecDT1 was confirmed when LysoPC was the substrate (for SM, no difference was observed for the action of the three PLDs) (Figure 5A). The densitometry of bands from phospholipid substrates and generated products confirmed such results and revealed that, while LgRecDT1 degraded 100% of LysoPC, LiRecDT1 cleaved 80% of this substrate and LlRecDT 87% (Figure 5B).

**Figure 6:**
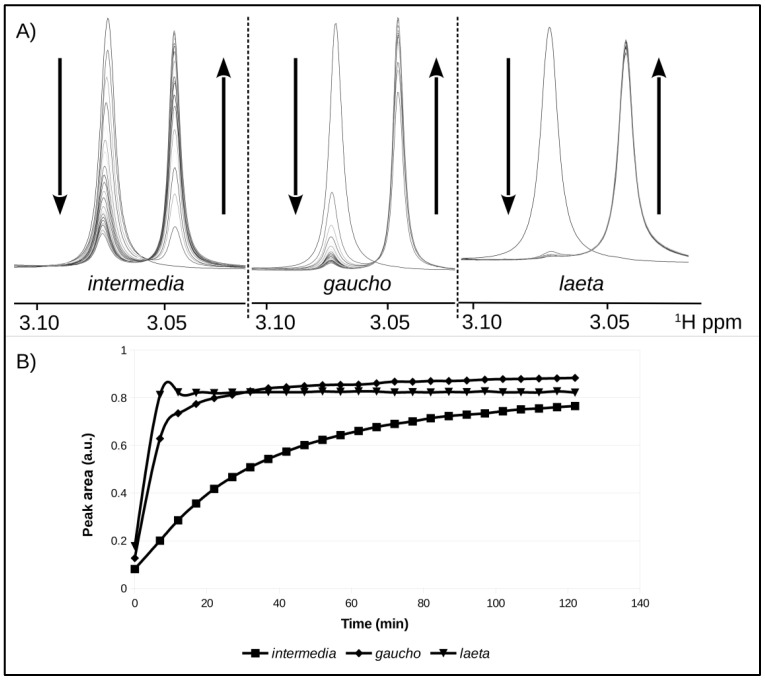
In situ time-dependent analysis NMR evaluation of products obtained following cleavage of SM after incubation with recombinant PLDs. (**A**) NMR signals of SM and the product formation after incubation with LiRecDT1 (*intermedia*), LgRecDT1 (*gaucho*), and LlRecDT1 *(laeta*). Arrows pointing up represent product formation in the ppm scale in the NMR spectra, while arrows pointing down represent cleavage of the substrate. ^1^H ppm is indicated on the *X*-axis. (**B**) Quantification of the product formation in the time course (0 to 140 min). The areas of the NMR signals from products were calculated and are proportional to the amount of product formed.

Comparative NMR analysis of reactions with sphingomyelin (the phospholipid substrate that is more rapidly degraded) incubated with the recombinant PLDs was conducted. As degradation of the substrate takes place, the intensity of the signal at δ 3.08 ppm, which corresponds to intact SM decreased continuously, and a signal at δ 3.05 ppm, which corresponds to the generated products, were detected. As the area under the 1H-NMR signal is proportional to the concentration, it was used to accurately quantify the products generated in a time course. Figure 6A,B compares the catalytic rates of each recombinant PLD during the time course of 120 min. As presented, LlRecDT1 possessed the highest catalytic rate towards SM, and, as shown in the first spectrum, 7 min after the addition of the enzyme, most of the product had been converted to approximately 82% catalysis. Similarly, LgRecDT1, the PLD from *L. gaucho* venom, also demonstrated a high catalytic rate, and after 27 min, a similar amount of product was generated as by LlRecDT1. The NMR data revealed that LiRecDT1, the recombinant PLD from *L. intermedia* venom, has a lower catalytic rate when compared to the other enzymes, as after 120 min of incubation with SM, it exhibited approximately 76% catalysis. Thus, the PLDs from *L. gaucho* and *L. laeta* possess a higher degrading activity upon SM than the PLD from *L. intermedia* venom (Figure 6B).

### 2.5. Hemolytic Activity

**Figure 7:**
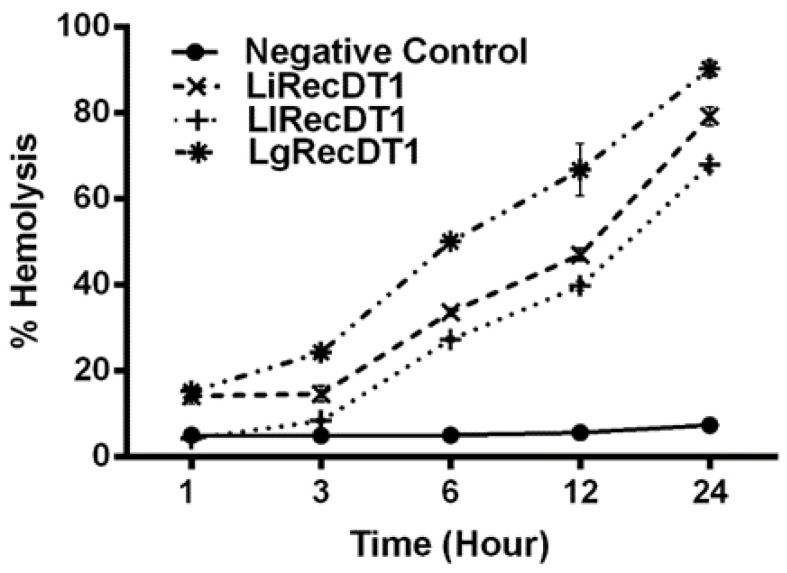
Evaluation of time-dependent hemolysis caused by recombinant brown spider venom PLDs. Human erythrocytes exposed to recombinant PLDs (5 µg/mL) at 37 °C were assayed for lysis quantification from 1 to 24 h. The absorbances of the supernatants were measured at 550 nm and the percentages of hemolysis were calculated by comparing the absorbance values induced by 0.1% *v/v* Triton X-100 (100%). The supernatant of cells incubated with TBS was used as a negative control. Values represent an average of five experiments ± SEM.

We also evaluated recombinant PLDs using the direct hemolytic activity of these enzymes as a model, as the quantification of this effect as previously described [12]. Red blood cells were incubated with toxins and the free hemoglobin obtained as a product of lysis was quantified over time up to 24 h. The results depicted in Figure 7 show that all three toxins possess direct hemolytic activity; however, *L. gaucho* PLD has the highest hemolytic activity in all the intervals analyzed, reaching 93% of hemolysis in 24 h, followed by PLDs from *L. intermedia* (79% in 24 h) and *L. laeta* (75% in 24 h), but with not-significant statistical differences when compared to each other (Figure 7).

### 2.6. Animal Lethality

As the involvement of *Loxosceles* PLDs in mouse lethality has already been described, we evaluated the median lethal dose (LD_50_) of recombinant PLDs using mice as the animal model. Mice were inoculated intraperitoneally with three different amounts of toxins, and the death/survival rates were observed for up to 48 h. Table 1 shows these rates and percentages of mortality according to the doses and time intervals. LgRecDT1, the PLD from *L. gaucho*, was the most lethal toxin with an LD_50_ of approximately 12.5 µg between 28 and 30 h after exposure. In addition, all animals treated with different concentrations of LgRecDT1 died between 24 h and 42 h after exposure to 12.5 µg, while no animal died in this interval when inoculated with LiRecDT1 or LlRecDT1. LlRecDT1 presented an LD_50_ of approximately 25 µg between 28 and 30 h after PLD exposure. All mice treated with 50 µg of LlRecDT1 died 30 h after toxin exposure, and no mouse died when treated with 12.5 µg following 48 h of exposure. LiRecDT1 was the less lethal enzyme when compared to the other two tested enzymes as it displayed an LD_50_ of approximately 50 µg between 28 and 30 h after inoculation, and no mouse died when treated with 12.5 µg and 25 µg even after 48 h of exposure.

### 2.7. Insecticidal Activity of Recombinant PLDs

**Figure 8:**
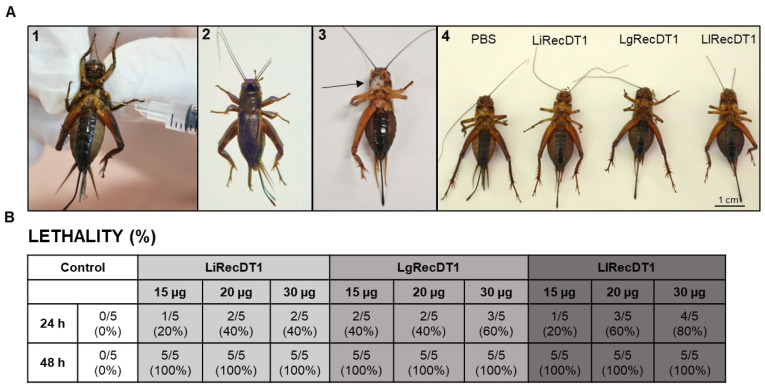
Lethality of PLDs on crickets. (**A**) Images of experiments. (1) Site of application of the toxins, which was between the second and third pair of legs in the ventral region of the animal. (2) Crickets treated with PBS (negative control). (3) Representative crickets treated with one of the PLDs after approximately 45 min. It is possible to observe several responses such as paralysis of the hind legs and a whitish secretion expelled through the mouth. (4) Representative animals from all experimental groups indicated swelling of the abdomen in animals inoculated with PLDs when compared to the negative control group (PBS). (**B**) The table depicts the death/survival rates and the percentage of crickets’ mortality in different amounts (15 µg, 20 µg, and 30 µg) after 24 and 48 h of exposition to LiRecDT1, LgRecDT1, and LlRecDT1.

The PLDs from Brown spider venoms may present insecticidal activity, as shown by [28]. We analyzed the insecticidal activity of the recombinant PLDs studied herein. Crickets were injected in the second segment of the abdomen with 15, 20, and 30 µg of PLDs and the death/survival rate was determined after 24 and 48 h. Phosphate-buffered saline (PBS) was used as the negative control. Figure 8 depicts the death/survival rates and the percentages of cricket mortality as a function of the dose and time intervals, and images present the insect behaviors following inoculation with PLDs. After 24 h of exposure to the toxin and at all concentrations tested (15 µg, 20 µg, and 30 µg), the PLDs of *L. laeta* and *L. gaucho* showed greater potential lethality and/or paralytic effects when compared to *L. intermedia*. After 48 h of toxin expositions, no insects survived.

### 2.8. Molecular Dynamics (MD) Simulations of PLDs and LysoPC Micelles

**Figure 9:**
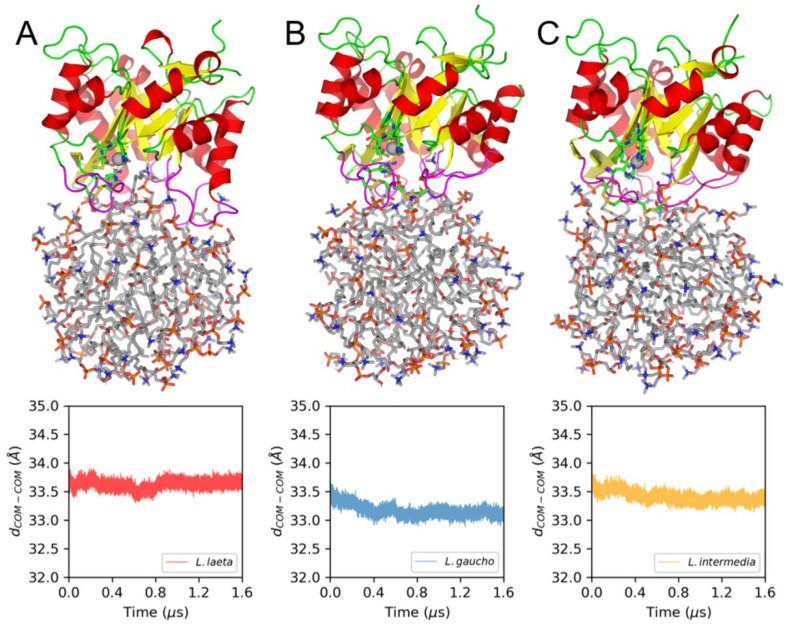
Microsecond-long MD simulations of PLDs bound to LysoPC micelles. (**A**) LlRecDT1, (**B**) LgRecDT1, and (**C**) LiRecDT1. The PLD-micelle structures represented in the figure correspond to the final frames generated in the respective 1.6 μs MD simulations. Proteins are presented as cartoons. Secondary structural elements are colored differently, i.e., α-helices in red, β-strands in yellow, and loops in green, except for the loops interacting with the micelles, which are depicted in magenta. Active site residues and Cys residues forming S-S bonds are represented as green sticks, and LysoPC molecules are shown as gray sticks. The lower panel of the figure shows the time profiles of the distance between the centers of mass of the PLD and the micelle (*d_COM-COM_*) during the MD simulations. All distances were calculated starting from the first frame of the productive runs.

Furthermore, 1.6 μs MD simulations were carried out for the three PLDs bound to LysoPC micelles starting from manually setup conformations in which the active sites of the enzymes were oriented toward the lipids. This orientation remained relatively unaltered, as can be inferred from the structural representations of the final frames collected from each MD simulation (Figure 9). Furthermore, the time profiles of distances between the centers-of-mass (*d_COM-COM_*) of the micelle and the bound PLD also show that the interaction between these molecules occurred during the whole MD simulation time in all cases (Figure 9). The protein–lipid interactions involving several loops flanking the PLD active sites (Figure 9A–C) will be further analyzed. 

The Molecular Mechanics Generalized Born Surface Area (MM-GBSA) effective free energy calculations were carried out for every PLD-micelle system based on multiple independent 400 ns MD simulations initiated from the frames collected during the respective 1.6 μs MD trajectories. The results presented in Table 2 indicate that there are significant differences in the interaction energies (∆*G_eff_*) of the PLDs with the LysoPC micelles. In fact, LlRecDT1 displayed the most favorable ∆*G_eff_* value, followed by LgRecDT1, with LiRecDT1 possessing a lower affinity for the micelle. The latter is in agreement with the activity assays showing that LiRecDT1 displayed the lowest catalytic rate against LysoPC among analyzed PLDs. As for the other two PLDs, the in silico results diverge with the experimental activities against LysoPC, thus indicating that other factors beyond the interactions with the lipids can be at play. Another interesting conclusion derived from the analysis of the free energy components is that the largest affinity of LlRecDT1 is caused by its relatively more favorable non-polar energies, which include van der Waals and non-polar solvation terms (Table 2). As the former terms become more negative, the number of contacts and the surface area of the complex become greater. Therefore, the previous results indicate that LlRecDT1 forms the most extensive interface with the LysoPC molecules among the three PLDs analyzed here.

**Figure 10:**
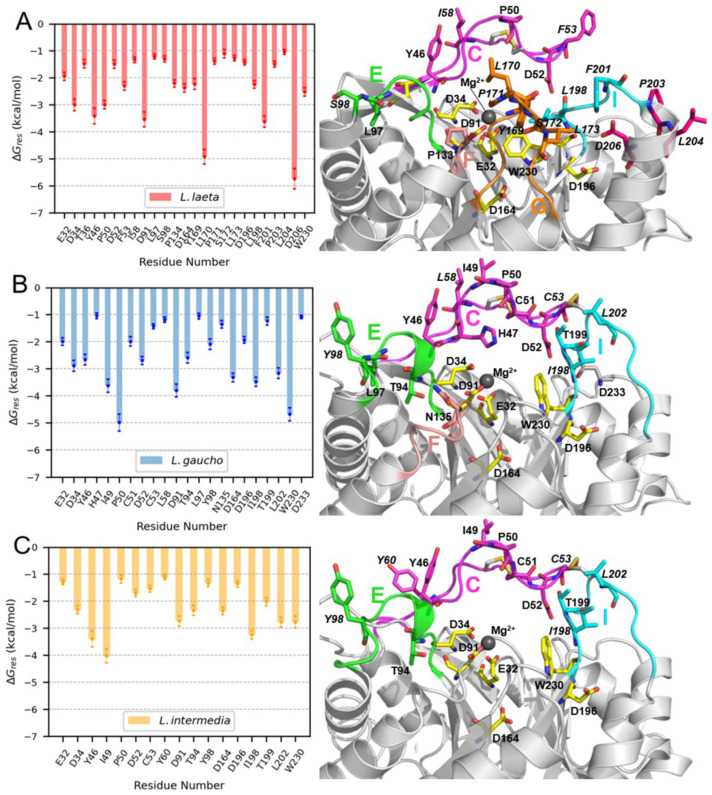
Hotspot residues of PLDs for the interaction with LysoPC micelles. (**A**) LlRecDT1, (**B**) LgRecDT1, and (**C**) LiRecDT1. Graphs on the left show the per-residue energy contribution to the binding affinity (∆*G_res_*) for hotspot residues (∆*G_res_* ≤ −1 kcal/mol) for each PLD. On the right, the represented structures highlight the hotspot residues. Residues are colored according to the region of the protein where they are located, with active site residues lying in the central β-barrel being depicted in yellow, and residues in the loops being colored in magenta (loop C), green (loop E), salmon (F), orange (loop G), and cyan (loop I). Residues in α-helices are colored in dark pink. Non-conserved residues are labeled in italic. The single letter loop nomenclature corresponds to that used by [20].

As indicated in Figure 9, variations are noted in the active site-flanking loops of the PLDs that interact with the LysoPC micelles. To determine the relevance of the residues located in these loops and in other regions, we conducted per-residue free energy decomposition of the effective binding free energy. The results obtained by this computational approach confirmed that most of the hotspot residues are located within these active site-flanking loops, especially loops C and I of the three PLDs and loop G exclusively in LlRecDT1 (Figure 10). Some active site residues, such as E32, D34, and D91, which coordinate the Mg^2+^ ions and W230 were also predicted to be among the residues that are involved in the binding of lipids. LlRecDT1 possesses the largest number of hotspot residues, which is consistent with its more favorable affinity for LysoPC micelles, and, differently from the other PLDs, its loop G residues (Y169, L170, P171, S172, and L173) are actively involved in the formation of the protein–lipid interface. Moreover, only in LlRecDT1, do we observe the presence of hotspots pertaining to an α-helix (α6), with one of its residues, D206, displaying the largest ∆*G_res_* value for this protein. Although LiRecDT1 and LgRecDT1 show great structural similarity in the region comprising the active site and flanking loops, we also found noticeable differences in the pattern of hotspot residues in both PLDs. The number of hotspots in LgRecDT1 is greater than in LiRecDT1, suggesting a stronger interaction between the former protein and the LysoPC micelles. Most of these differences are concentrated in loop C, which seems to form more stable interactions with the lipids in LgRecDT1 than in LiRecDT1. 

**Figure 11:**
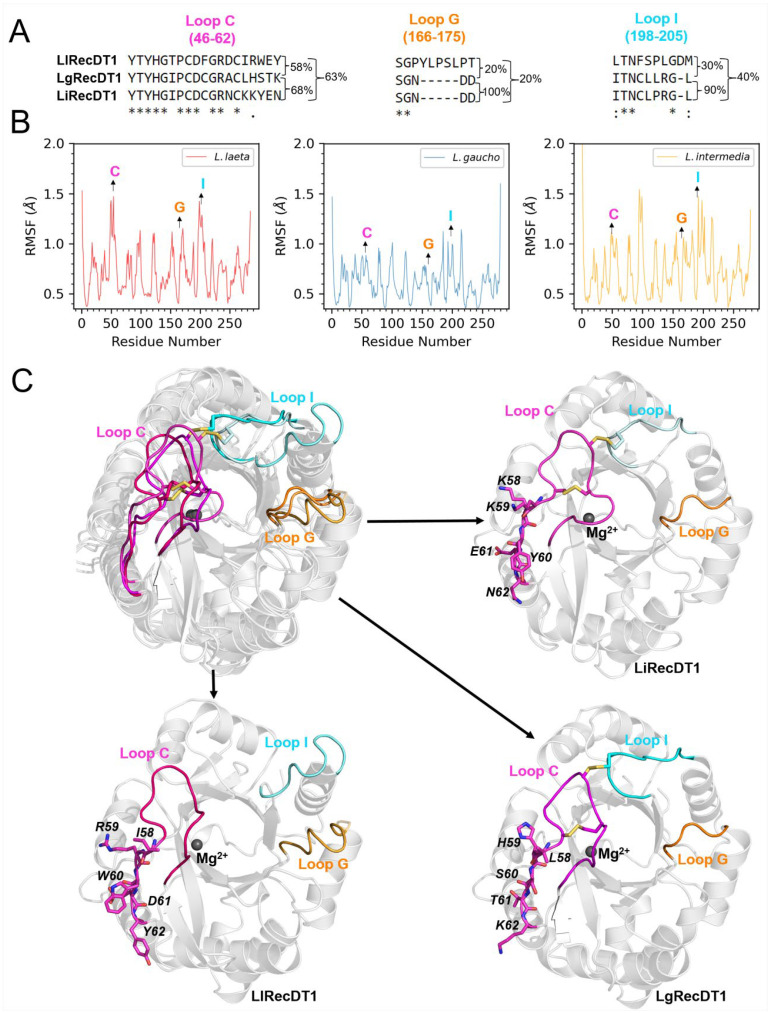
Main conformations of the active site-flanking loops C, G, and I adopted upon interacting with LysoPC micelles. (**A**) Aligned sequences of the loops in the three analyzed PLDs. The pairwise sequence identities are shown on the right for all loops. Consensus symbols are used to indicate the occurrence of identical residues (*), conserved substitutions (:) or semi-conserved substitutions, i.e., residues with similar shapes (.) at a given position. (**B**) Root mean square fluctuation (RMSF) profiles for the three enzymes with respect to the backbone atoms during the concatenated 400 ns MD simulations. Regions corresponding to loops C, G, and I are indicated. (**C**) Structural representations of the main conformations of the analyzed PLDs obtained by clustering the concatenated MD simulations conducted for each PLD-micelle system. Clustering was performed with respect to the heavy atoms of the residues lying in the three highlighted loops. Non-conserved residues of the segment 58–62 are shown in sticks. The three structures were superimposed (upper left) and depicted individually. Lipids are removed for clarity. Equivalent loops in different proteins are shown in different shades of the colors used in (**A**,**B**) to identify each loop.

To improve our understanding of the differential role that active site flanking loops C, G, and I (Figure 11A) are likely to play in the interaction of PLDs with micelles, we analyzed the flexibility of the proteins, particularly in the regions of these loops, along with the PLD-micelle MD simulations (Figure 11B). Loop C possessed greater flexibility in LlRecDT1, a feature that can be attributed to the lack of an S-S bond linking loops C and I, which can restrict their relative displacement. LgRecDT1 displayed an overall decrease in the flexibility of loops interacting with the LysoPC micelle in comparison with LiRecDT1. This result reinforces our earlier conclusion that despite the structural similarities between LiRecDT1 and LgRecDT1, their specific interactions with LysoPC micelles are distinct. In Figure 11B, the principal conformations of the active site-flanking loops in the three analyzed PLDs when bound to the LysoPC micelles are different. Such conformational differences are expected for LlRecDT1, since, only in this PLD, loop I is detached from loop C, and loop G is longer than in the other enzymes (Figure 11A,C). However, loop C, which has a relatively high degree of conservation in the three PLDs, demonstrated significant conformational differences (Figure 10C). This is likely to stem from the divergence in sequences at the C-terminal region of the loop, comprising residues 58 to 62. Interestingly, residue 58 was predicted as a hotspot for the interaction of LlRecDT1 (L58) and LgRecDT1 (I58) with the LysoPC micelle (Figure 10A,B), whereas K58 of LiRecDT1 has an unfavorable contribution to the lipid binding (∆*G_res_* = 0.7 ± 0.2 kcal/mol), thus reinforcing the model that loop C might play a key role in determining the differences in the predicted affinities of the PLDs for the micelles (Table 2).

## 3. Discussion

### 3.1. General Discussion

Brown spiders of the *Loxosceles* genus possess two venom glands that are able to synthesize, store, and secrete several molecules with distinct biological properties [2]. Previous studies have attempted to correlate the biochemical and biophysical properties of venom proteins with their in vitro activities [4,15]. Transcriptomes of *Loxosceles* venom glands [29,30,31,32] and proteomes of venoms [33,34,35] have identified numerous proteins with varied toxic actions upon different species. The PLDs are particularly interesting since they reproduce in vitro several manifestations of loxoscelism, such as dermonecrosis, with the presence of abundant inflammatory polymorphonuclear cells and a prominent edema. Systemic signals such as hemolysis, thrombus formation inside blood vessels, platelet aggregation, and acute renal failure can also be reproduced following the treatment of animals with recombinant PLDs [10,11,12,24]. Understanding the underlying molecular and cell signaling pathways triggered by these enzymes, particularly those implicated in uncontrolled inflammatory response is of relevance [23,25,36,37,38,39,40]. Comparative analyses of transcripts from the venom glands of different brown spider species identified the variations in toxin contents for the Brazilian *L. laeta* [29], Peruvian *L. laeta* [32], *L. similis* [31], and *L. intermedia* [30]. Venom proteome analyses of *L. gaucho* [34] and *L. intermedia* [33,35] also demonstrated the existence of differences in the toxin profiles. Comparative gender and species variation analysis demonstrated that certain biological activities were more prominent in the venoms of female spiders, especially from *L. laeta* [41].

In South America, most accidents with Loxosceles spiders are caused by three species: L. intermedia, L. laeta, and L. gaucho [3,4,7] designated as LiRecDT1, LlRecDT1, and LgRecDT1 (Loxoceles intermedia/laeta/gaucho Recombinant Dermonecrotic Toxin) wherein the term “Dermonecrotic Toxin” is with reference to the necrotic lesions caused by these PLDs in the skin of victims and in animal models [4]. The production of recombinant wild-type and mutant *Loxosceles* PLDs has been important for the structural and biological studies of these enzymes [11,21,23,42], with the aim of understanding the observed differences in the activities at the molecular level.

### 3.2. Sequence Identity/Structure

*Loxosceles* PLD amino acid sequences are highly conserved, presenting between 55% and 99% of identity, with calculated molecular masses varying from 31.22 kDa to 32.79 kDa and isoelectric points ranging from 5.3 to 8.7. CD spectral analysis [21,41] and crystal structures indicate these proteins possess homologous structures referred to as the TIM-barrel or (α-β)_8_barrel [16,17,18]. All the amino acid residues involved in hydrolysis, magnesium ion coordination, and substrate binding are fully conserved in LiRecDT1, LlRecDT1, and LgRecDT1. These PLDs are classified based on the number of disulfide bridges present in their structures. The catalytic loop (loop C) of the PLDs is stabilized by a conserved disulfide bond formed between C51 and C57. LlRecDT belong to class I PLDs and have only this disulfide bond. LiRecDT1 and LgRecDT1 are class II *Loxosceles* PLDs, which additionally possess a second disulfide bridge formed between C53 and C201, which links the catalytic loop (loop C) with the flexible loop (loop I) [17,18,19]. 

Differences were observed in thermostability only at higher temperatures: LiRecDT1 and LgRecDT1 presented a similar DSC profile and melting temperature values (60 °C and 59 °C, respectively), whereas the melting temperature of LlRecDT1 was lower (51 °C). This difference may be due to the presence of only a single intra-chain disulfide bridge (C51–C57) in the *L. laeta* PLD, which leads to an increase in the molecular flexibility and a decrease in thermostability, and site-directed mutations C53A-C201A reduced the melting temperature of LiRecDT1, supporting these results [21].

Immunoassays using polyclonal antisera against purified PLDs revealed antigenic cross-reactivity among the studied PLDs, suggesting the presence of similar sequence/epitopes and confirming the structural conservation of PLDs in *Loxosceles* venoms [30,33,34,43]. However, the antibodies to LgRecDT1 and LlRecDT1, besides producing intense reactions with homologous antigens, also cross-reacted more strongly with the other two PLDs when compared to the polyclonal sera produced against LiRecDT1, indicating that structural differences and different antigenic determinants are responsible for the observed differences in immunogenicity and functionality.

### 3.3. Membrane Binding Mode

These PLDs hydrolyze SM, producing ceramide 1-phosphate or cyclic lipids and choline, and are also able to cleave lysoglycerophospholipids and lyso-platelet-activating factor (LPAF) to produce the lipid mediator lysophosphatidic acid (LPA) or cyclic products [22,23,24]. Fluorimetric quantification of choline, HPTLC detection of substrates/products, and NMR spectroscopy detection of substrates/products demonstrated that the three isoforms cleaved SM and LysoPC at different velocities in a time-dependent manner. LgRecDT1 was able to completely degrade LysoPC (100%) when compared to the other toxins (LiRecDT1—80% and LlRecDT1—87%). The NMR results revealed significant differences in the enzymatic activities of LgRecDT1 and LlRecDT1 (Figure 6) and indicate that LgRecDT1 and LlRecDT1 possess higher catalytic activity than LiRecDT1. 

These PLDs play a significant role in the hematologic disturbances observed in systemic loxoscelism, such as platelet aggregation, thrombus formation in blood vessels, intravascular hemolysis, and hemolytic anemia [12,13,44,45]. The hemolytic activity of PLDs can be direct, acting directly upon erythrocytes’ membrane, and/or indirect, activating the complement system. Some recombinant *Loxosceles* PLDs possess hemolytic activity triggered by both mechanisms. These two mechanisms may contribute to the intravascular hemolysis and hemolytic anemia observed after some cases of loxoscelism [12,40]. Lipid analysis of human red blood cells membrane indicates a ratio of 19.5% of sphingomyelin, 2.7% of lysophosphatidylcholine, and 35.5% of phosphatidylcholine [46], which are substrates for brown spider PLDs; LgRecDT1 presented a higher in vitro hemolytic activity after 24 h (93%) when compared to LlRecDT1 (79%) and LiRecDT1 (75%). This higher hemolytic activity of LgRecDT1 may be due to its higher enzymatic activity upon phospholipid substrates.

The in vivo effect in mice lethality showed that LgRecDT1 is the more lethal toxin (LD_50_ of approximately 12.5 μg) when compared to LlRecDT1 (LD_50_ of approximately 25 μg) and LiRecDT1 (LD_50_ of approximately 50 μg). In addition, in vivo experiments were also carried out to study insecticidal activity using crickets. Zobel-Thropp et al. [28] evaluated the insecticidal activity of a recombinant PLD from *Loxosceles arizonica* in crickets. The authors showed that the recombinant PLD is more toxic for crickets than the crude venom of *L. arizonica*. In both treatments, crickets did not recover from the paralysis and died. Herein, PLDs from the venoms of *L. gaucho* and *L. laeta* have more toxic activities upon these insects when compared to PLD from *L. intermedia*, which is in agreement with their functional differences assessed with in vitro protocols and corroborating it with mice lethality results. 

To understand the differences in the enzymatic activities in the PLDs from *L. laeta*, *L. gaucho*, and *L. intermedia* against SM and LysoPC in water, we modeled simple systems consisting of the enzyme and a small LysoPC micelle and conducted molecular dynamics (MD) simulations and free energy calculations. In the structural models used for these simulations, the three extended loops, the catalytic loop (C), flexible loop (I), and the variable loop (G), which protrude from the torus of the *(α-β)*_8_ barrel, mediate binding to LysoPC micelles. The results demonstrated that LlRecDT1 possesses the highest affinity for LysoPC micelles, followed by LgRecDT1, and then LiRecDT1, which is in correlation with the observed trends in the catalytic assays. Free energy calculations, H^1^-NMR, and Amplex Red assays were unable to capture the differences in the activities of LgRecDT1 and LlRecDT1 against LysoPC. These differences in the binding affinity for lipid aggregates are likely to be key factors in determining the overall catalytic rates. The more accessible active site of LlRecDT1, due in part to the absence of the second S-S bridge linking loops C and I, seems to be relevant for the higher affinity for the micelles. Differences in the amino acid compositions of the C-terminal fragments of loop C in LgRecDT1 and LiRecDT1 may also be important for lipid binding. A complete mechanistic study of the activity of PLDs must encompass the calculation of kinetic constants, translocation rates of LysoPC molecules from the micelle into the active site, and product release. 

## 4. Materials and Methods

### 4.1. Reagents

Polyclonal antibodies against recombinant PLDs from *L. intermedia*, *L. laeta*, and *L. gaucho* venoms were produced in mice as described previously [47]. Five animals were immunized with each PLD, and the pools were used in the subsequent assays to avoid distortions from individual immunological responses. NaCl and KCl were purchased from Merck (Darmstadt, Germany). Tryptone and yeast extract were obtained from Himedia (Mumbai, India). Chloramphenicol and ampicillin were purchased from USB Corporation (Cleveland, OH, USA). SM (sphingomyelin egg, chicken) and LysoPC (l-α-lysophosphatidylcholine egg, chicken) were purchased from Avanti Polar Lipids, Inc. Alabaster, AL, USA. Coomassie Blue dye, Tris, and sucrose were obtained from Bio-Rad (Hercules, CA, USA). Xylazine and ketamine were purchased from Rhobifarma (Hortolândia, São Paulo, Brazil).

### 4.2. Recombinant Protein Expression and Purification

The constructs of LiRecDT1 from *L. intermedia* (GenBank: DQ218155.1), LgRecDT1 from *L. gaucho* (LgRec1—GenBank: JX866729.1), and LlRecDT1 from *L. laeta* (SMase I—GenBank: AY093599.1) were obtained as described previously [9,48]. Proteins were expressed in BL21(DE3)pLysS *Escherichia coli* cells as described earlier [9,48]. The cells were thawed and disrupted by mechanical lysis. The lysed samples were centrifuged (9000× *g*, for 30 min, at 4 °C), and the supernatants were purified on an Äkta pure system (GE Healthcare Life Sciences) using a Histrap™ HP column (Cytiva, Uppsala, Sweden). Briefly, the samples (filtered in 0.22 µm) were equilibrated with buffer A (20 mM NaH_2_PO_4_, 500 mM NaCl, pH 7.5) and washed with 5 mL of 4% of buffer B (20 mM NaH_2_PO_4_, 500 mM NaCl, 500 mM Imidazole, pH 7.5), and the proteins were eluted with a linear gradient of buffer B. The samples were collected and dialyzed thrice against a phosphate-buffered saline (PBS) buffer and analyzed on 12.5% SDS-PAGE gels.

### 4.3. Immunological Cross-Reactivity

Protein quantifications were performed by the Coomassie Blue method according to the procedures outlined by Bradford [49]. Protein samples of recombinant toxins (5.0 µg) were analyzed by 12.5% SDS–PAGE under reduced conditions with β-mercaptoethanol (5%). The toxins were transferred onto nitrocellulose membranes for immunoblotting and incubated with hyperimmune serum (pools of five animals) raised against either *L. intermedia, L. laeta,* or *L. gaucho* recombinant PLDs D (1:2000). This was followed by detection using secondary alkaline phosphatase-coupled anti-IgG (1:8000) (Sigma-Aldrich, St. Louis, MO, USA) and visualization of immunoreactions through the BCIP/NBT substrate reaction (Promega, Madison, WI, USA). Control of primary antibody specificity was performed using pre-immune sera (collected before immunization). Immunoblotting reactions were quantified by means of densitometry determinations of the positive bands by using the Image J Analysis Software 1.54f. The reactions of the antibodies with the recombinant PLDs from the same species were normalized to 100% to calculate the percentages of cross-reactions with recombinant PLDs from other species. Additionally, an ELISA-antibody capture assay was performed by coating the plates (Nunc MaxiSorp, Roskilde, Denmark) with recombinant PLDs (20 μg/mL) overnight at 4 °C. Primary reactions were developed for 2 h with hyperimmune sera against PLDs. Secondary reactions used HRP-conjugated antibodies for 1 h at room temperature. The colorimetric reaction was developed using orthophenylene diamine (OPD) and the substrate H_2_O_2_ in a citrate buffer [47]. Absorbance values were determined at 492 nm with a Meridian ELX 800 spectrophotometer. All measurements were performed in pentaplicate.

### 4.4. Circular Dichroism Spectroscopy (CD)

Recombinant LiRecDT1, LlRecDT1, and LgRecDT1 were dialyzed at 4 °C against a phosphate buffer (10 mM NaH_2_PO_4_/Na_2_HPO_4_ at pH 7.4) to final concentrations of 0.5 mg/mL. The spectra were obtained by using a Jasco J-810 spectropolarimeter (Jasco Corporation, Tokyo, Japan) using a cuvette with a 1 mm path length. Each spectrum (collected at 0.5 nm intervals) was the average of eight measurements, performed at a rate of 50 nm/min, a response time of 8 s, and a bandwidth of 1 nm. The temperature was maintained constant at 20 °C [21]. Deconvolution was performed using the K2D2 tool [50,51].

### 4.5. Differential Scanning Calorimetry (DSC)

DSC experiments were performed using N-DSC III (TA Instruments, New Castle, DE, USA) in the temperature ranges of 20–90 °C with a heating and cooling rate of 1 °C/min. The proteins (LiRecDT1, LlRecDT1, and LgRecDT1) were analyzed by dilution in a phosphate buffer (10 mM NaH_2_PO_4_/Na_2_HPO_4_, pH 7.4) to a final concentration of 1.0 mg/mL. Both calorimeter cells were loaded with the buffer solution, equilibrated at 10 °C for 10 min, and scanned repeatedly as described above until the baseline was stable and reproducible. The sample cell was subsequently loaded with each protein separately and scanned in the same manner. The baseline correction was conducted by subtracting the ‘buffer vs. buffer’ scan from the corresponding ‘protein vs. buffer’ scan.

### 4.6. Amplex Red^TM^ Assay

The enzymatic activities of recombinant PLDs upon sphingomyelin and lysophosphatidylcholine were measured using the Amplex Red Assay Kit (Molecular Probes (Eugene, OR, USA), Invitrogen (Waltham, MA, USA)). In this assay, enzymatic activity was monitored using 10-acetyl-3,7-dihydroxyphenoxazine (Amplex Red reagent), a sensitive fluorogenic probe for H_2_O_2_. The degradation of lipid substrates yielded choline as one of the products. Choline was then oxidized by choline oxidase to betaine and H_2_O_2_. In the presence of horseradish peroxidase, H_2_O_2_ reacted with the Amplex Red reagent in a 1:1 stoichiometry to generate the highly fluorescent product resorufin [14]. LiRecDT1, LlRecDT1, and LgRecDT1 (2.5 µg each, in five trials) were added to the Amplex Red reagent mixture containing 250 μM (in 0.01% Triton X-100) of the substrates (egg sphingomyelin and l-α-lysophosphatidylcholine (egg lyso PC) from Avanti Polar Lipids, Inc. Alabaster, AL, USA). The negative control was obtained by incubation of the Amplex Red reagent mixture in the absence of toxins. The reaction tubes were incubated at 37 °C for 1 h, and fluorescence was measured in a microplate fluorimeter (Tecan Infinite^®^ M200, Männedorf, Switzerland) using excitation and emission detection wavelengths of 540 nm and 570 nm, respectively.

### 4.7. HPTLC (High-Performance Thin-Layer Chromatography) 

To analyze the direct enzymatic activity by PLDs, 25 µg of LiRecDT1, LlRecDT1, and LgRecDT1 were incubated for 60 min with 50 µg of egg sphingomyelin or l-α-lysophosphatidylcholine in 2% Triton X-100. All samples were recovered directly by partition with water-saturated 1-butanol and the butanol fraction and were dried. Lipid samples were suspended in chloroform and 20 µL of each resultant solution was applied to silica gel 60 plates (Merck) using 40% chloroform:methanol:methylamine (65:35:10 *v*/*v*/*v*) as the mobile phase. The HPTLC samples were then visualized after being sprayed with molybdenum blue [21]. Differences in lipid degradation following enzyme treatments were quantified by densitometry of the digital images of HPTLC plates, acquired with the GeneSnap software 7.1 for G: Box Chemi XL (Syngene, Cambridge, UK) and quantified by the Quantity One software 4.6 for Chemi Doc XRS (Bio-Rad, Hercules, CA, USA). After quantification, negative control bands were normalized to 100% to allow a comparison of the samples.

### 4.8. NMR Spectroscopy (Nuclear Magnetic Resonance)

Enzyme catalysis was monitored by nuclear magnetic resonance (NMR) [52,53]. The experiments were carried out in a 600 MHz (1H) Bruker Avance III HD spectrometer (Bruker, Mannheim, Germany) equipped with a triple-resonance of pulse field and z-gradient cryo-probe. 

A Tris-buffered solution, 20 mM and pH 7.4, was used to solubilize a previously prepared 5 mM sphingomyelin in 5% ethanol, 2% Triton X-100, 100 mM Tris-HCl, and 10 mM MgCl_2_, pH 7.4, resulting in 1 mM sphingomyelin solution. The solution was transferred to 3 mm NMR tubes, containing 10% D_2_O, and an NMR spectrum was collected using excitation sculpting for water suppression [54]. Experiments were collected using 32 scans (4 dummy scans), a recovery delay of 100 μs, with a square pulse of 2 ms, two sine-shaped gradients of 31 and 11%, and an homospoil pulse of 1000 μs for excitation sculpting. Then, PLDs were added to a final concentration of 1 μM, gently mixed in a vortex for 30 s, and returned to the NMR spectrometer to monitor the sphingomyelin signal in a series of experiments for 120 min. The first experiment after adding the enzyme was collected after 7 min and all the subsequent experiments in a time delay of 5 min.

### 4.9. Hemolysis Assay

The hemolysis experiment was performed as previously described [12]. Washed human red blood cells (1 × 10^8^ cells) were added to microtubes containing 25 µg/mL (10 µg) of LiRecDT1, LlRecDT1, or LgRecDT1 in Tris Buffer Sucrose (TBS: 250 mM sucrose; 10 mM Tris/HCl, pH 7.4). Red blood cells in TBS served as the negative control, and for the positive control, red blood cells were incubated in 0.1% *v/v* Triton X 100. Experiments were performed in pentaplicate, and incubation was performed with gentle agitation at different intervals (0, 3, 6, 12, and 24 h). Subsequently, the controls and samples were centrifuged for 3 min at 350× *g* using a refrigerated microfuge (Centrifuge 5804 R, Eppendorf, Hamburg, Germany), and supernatants were measured immediately at 550 nm (Meridian ELx 800, BioTek Instruments, Winooski, VT, USA). Absorbance was converted to percentage hemolysis considering the absorbance of the positive control (Triton X-100) as 100% hemolysis.

### 4.10. Animals

Adult Swiss mice (25–30 g) from the Central Animal House of the Federal University of Parana were randomly selected for in vivo experiments. Crickets were purchased from “Bicho Brasileiro Tiziu”, Curitiba, Parana, Brazil. All procedures involving vertebrate animals were performed in accordance with Brazilian Federal Laws, in accordance with the Ethical Subcommittee on Research Animal Care Agreement number 1467 of the Federal University of Parana.

### 4.11. Lethality

Purified recombinant toxins (12.5, 25, and 50 μg/kg) were injected intraperitoneally into five mice. Mice were observed for 8, 16, 24, 48, and 72 h after injection, and survival was assessed at 1 h intervals. PBS was used as negative control. For the insecticidal activity assays, recombinant PLDs were injected in the second segment of the abdomen of adult crickets at concentrations of 15, 20, and 30 μg/animal. Crickets were observed for 24 h after injection. PBS was used as the negative control.

### 4.12. Molecular Dynamics (MD) Simulations of PLDs ad LysoPC Micelles

To assess the ability of PLDs from *L. laeta* (LlRecDT1), *L. intermedia* (LiRecDT1), and *L. gaucho* (LgRecDT1) to interact with lipid substrates at the lipid–water interface through MD simulations, solvated systems containing a micelle and a PLD were prepared. Micelles containing 50 molecules of 1-myristoyl-2-hydroxy-*sn*-glycerol-3-phosphocholine (14:0 Lyso PC) were created using Charmm-GUI [55]. The initial configuration of the micelle was prepared for MD simulations using *tleap* of Amber22 [56]. The parameters of 14:0 LysoPC were derived from the Lipid 21 force field [57] with the *“--parametrize”* option of *packmol-memgen* [56,58]. The micelle was embedded in an octahedral box with edges of 10 Å from the micelle surface, filled with OPC water molecules and sufficient Na^+^ counterions to neutralize the system. The micelle solvated in water was subjected to 1000 cycles of unrestrained energy minimization (EM), with the first 5000 cycles corresponding to steepest descents (SD) EM and the final 5000 cycles to conjugate-gradient (CG) EM. Subsequently, the system was heated for 500 ps using a linear temperature gradient from 10 to 300 K and the Berendsen weak coupling algorithm [59]. Random initial velocities derived from the Maxwell–Boltzmann distribution at 10 K were assigned to the system atoms. Heating was followed by a 500 ps MD simulation at *T =* 300 K and *p* = 1 bar, with the pressure being controlled with the Berendsen barostat [59]. During the previous two equilibration steps, harmonic restraints of 10 kcal∙mol^−1^∙Å^−2^ were placed on all LysoPC heavy atoms. Finally, a 200 ns NPT MD simulation was conducted, using the Langevin thermostat and the Monte Carlo barostat [60] to maintain constant temperature and pressure at 300 K and 1 bar, respectively. 

The final configuration of the micelle generated during the 200 ns MD simulation was utilized as the initial structure to build the PLD-micelle systems. The crystal structures of LiRecDT1 (PDB: 3RLH), LlRecDT1 (PDB: 2F9R), and the Alpha-Fold2 [61] model of LgRecDT1 were protonated at the H++ webserver [62]. Subsequently, LiRecDT1 was manually placed on the surface of the LysoPC micelle using Pymol v2.3.0 [63]. The active site was oriented toward the lipids, as this is the binding mode required to ensure enzymatic activity. The two other PLDs were structurally aligned with LiRecDT1 so that the initial positions of all the studied PLDs were similar. The protein-micelle systems were placed in octahedral boxes with edges at least 10 Å away from the surface of the solutes. OPC water molecules [64] were added to the simulation boxes using *tleap* of Amber22 [56]. The protein was parametrized with the ff19SB force field, and the LysoPC molecules were treated as previously described. The solvated protein-micelle systems were subjected to EM, heating, and pressure equilibration, following the same protocol presented earlier for the micelle in water. Then, 1.6 μs NPT MD simulations at 300 K and 1 bar were run for every system. Finally, 50 evenly spaced frames from each of the 1.6 μs trajectories were collected after 100 ns and stripped off the water molecules to generate new protein-micelle starting configurations after being re-solvated with *tleap*. The same EM and equilibration steps described earlier were employed for all these new systems, and 400 ns productive MD simulations were then performed, totaling 2 μs of concatenated simulation time for each PLD bound to a LysoPC micelle. All productive MD simulations mentioned here were run with *pmemd.cuda* [56] using hydrogen mass repartitioning, which enabled the increase of the timestep from 2 to 4 fs [65].

### 4.13. MM-GBSA Free Energy Calculations

The 50 replicas of 400 ns MD trajectories generated per PLD-micelle system were employed for molecular mechanics generalized Born surface area (MM-GBSA) free energy calculations with the MMPBSA.py program [66]. For these calculations, the first 80 ns of each trajectory were discarded and a time interval of 800 ps between the frames was set. Effective binding free energies (∆*G_eff_*) were estimated for each replicate MD simulation using the GB-neck implicit solvation model [67] and averaged in order to predict the final value for each system. All other parameters related to the MM-GBSA calculations were set to their default values in Amber22 [56]. Standard errors of the mean (SEMs) were calculated by dividing the standard deviation of the ∆*G_eff_* values by the square root of the number of independent MD simulations (50).

In order to assess the influence of individual PLD residues on the interaction with the micelles at the lipid–water interface, per-residue free energy decomposition was carried out with MMPBSA.py program [66]. These calculations were performed using the same implicit solvation model (GB-neck) and followed the general setup described in the previous paragraph [67]. Hot-spot residues for micelle binding were defined as those whose per-residue free energy contribution (∆*G_res_*) was ≤−1 kcal/mol.

### 4.14. Trajectory Analyses

Distances, root mean square fluctuation (RMF), and clustering analyses for the generated trajectories were performed with *cpptraj* of Amber22 [56,68]. For clustering, the average linkage algorithm was employed and a predefined number of 5 clusters were generated for every system. The central structure of the largest cluster, which in all cases comprised more than 85% of the trajectories, was selected for representation.

### 4.15. Statistical Analyses

Statistical analysis of enzymatic activity data was performed using analysis of variance (ANOVA) and the Tukey test for average comparisons. ELISA and hemolysis analysis were performed using the GraphPad InStat program version 6.01 for Windows 7 Professional and the results were expressed as means ± SEM. Significance was determined as *p* ≤ 0.05.

## 5. Conclusions

Using recombinant PLDs expressed in the *E. coli* system, we obtained results that indicate that although PLDs from *Loxosceles* spider venoms share high sequence identity and amino acid residues in the catalytic and magnesium ion coordination sites, as well as an exosite where amino acid residues involved in the binding to phospholipid substrates are conserved, sequence and conformational differences of the surface loops are the principal determinants of the observed differences in their functional activities, immunological properties, and structural characteristics. The studied phospholipases D from *L. gaucho* and *L. laeta* venoms possess greater degrading activities upon sphingomyelin and lysophosphatidylcholine and a superior hemolytic effect for lethality on mice, insecticidal activity, and immunogenic properties when compared to phospholipase D from *L. intermedia*. Since phospholipases D are the toxins of *Loxosceles* venoms involved with complications in loxoscelism, these data indicate that accidents with species *L. gaucho* and *L. laeta* can generate more severe complications in patients, and clinicians from endemic regions for these species should remain alert. 

## Figures and Tables

**Table 1:** Effect of Brown spider venom recombinant PLDs on mice lethality.

	Control	LiRecDT1			LgRecDT1			LlRecDT1		
-	-	12.5 µg	25 µg	50 µg	12.5 µg	25 µg	50 µg	12.5 µg	25 µg	50 µg
16 h	0/5 (0%)	0/5 (0%)	0/5 (0%)	0/5 (0%)	0/5 (0%)	0/5 (0%)	0/5 (0%)	0/5 (0%)	0/5 (0%)	0/5 (0%)
20 h	0/5 (0%)	0/5 (0%)	0/5 (0%)	0/5 (0%)	0/5 (0%)	2/5 (40%)	4/5 (80%)	0/5 (0%)	0/5 (0%)	3/5 (60%)
24 h	0/5 (0%)	0/5 (0%)	0/5 (0%)	0/5 (0%)	2/5 (40%)	3/5 (60%)	5/5 (100%)	0/5 (0%)	1/5 (20%)	3/5 (60%)
30 h	0/5 (0%)	0/5 (0%)	0/5 (0%)	3/5 (60%) *	3/5 (60%) *	5/5 (100%)	-	0/5 (0%)	3/5 (60%) *	5/5 (100%)
36 h	0/5 (0%)	0/5 (0%)	0/5 (0%)	3/5 (60%)	3/5 (60%)	-	-	0/5 (0%)	3/5 (60%)	-
42 h	0/5 (0%)	0/5 (0%)	0/5 (0%)	4/5 (80%)	5/5 (100%)	-	-	0/5 (0%)	3/5 (60%)	-

* Points where each toxin reached a 3/5 death/survival rate are highlighted.

**Table 2:** MM-GBSA effective free energies for the interaction of PLDs with LysoPC micelles ^a^.

PLD	∆*G_El+GB_* ^b^ (kcal/mol)	∆*G_vdw_* ^c^ (kcal/mol)	∆*G_surf_* ^d^ (kcal/mol)	∆G*_eff_* ^e^ (kcal/mol)
LgRecDT1	76.3 ± 4.0	−129.7 ± 6.4	−17.2 ± 0.8	−70.6 ± 4.0
LlRecDT1	117.1 ± 5.3	−179.5 ± 8.0	−24.1 ± 1.0	−86.5 ± 4.1
LiRecDT1	98.3 ± 4.8	−136.3 ± 6.8	−18.8 ± 0.9	−56.7 ± 3.5

^a^ Mean value ±SEMs are reported. ^b^ Electrostatic free energy component including the polar solvation term calculated using the implicit solvation model. ^c^ Van der Waals free energy component. ^d^ Non-polar solvation-free energy component, which is proportional to the contact surface between the interacting molecules. ^e^ Total effective free energy.

## Data Availability

Data are contained within the article.
